# The Effect of Environment on the Evolution and Proliferation of Protocells of Increasing Complexity

**DOI:** 10.3390/life12081227

**Published:** 2022-08-13

**Authors:** Suvam Roy, Supratim Sengupta

**Affiliations:** Department of Physical Sciences, Indian Institute of Science Education and Research Kolkata, Mohanpur 741246, India

**Keywords:** origin of life, RNA world, protocell, ribozymes, primordial environment, hot spring hypothesis, evolution

## Abstract

The formation, growth, division and proliferation of protocells containing RNA strands is an important step in ensuring the viability of a mixed RNA–lipid world. Experiments and computer simulations indicate that RNA encapsulated inside protocells can favor the protocell, promoting its growth while protecting the system from being over-run by selfish RNA sequences. Recent work has also shown that the rolling-circle replication mechanism can be harnessed to ensure the rapid growth of RNA strands and the probabilistic emergence and proliferation of protocells with functionally diverse ribozymes. Despite these advances in our understanding of a primordial RNA–lipid world, key questions remain about the ideal environment for the formation of protocells and its role in regulating the proliferation of functionally complex protocells. The hot spring hypothesis suggests that mineral-rich regions near hot springs, subject to dry–wet cycles, provide an ideal environment for the origin of primitive protocells. We develop a computational model to study protocellular evolution in such environments that are distinguished by the occurrence of three distinct phases, a wet phase, followed by a gel phase, and subsequently by a dry phase. We determine the conditions under which protocells containing multiple types of ribozymes can evolve and proliferate in such regions. We find that diffusion in the gel phase can inhibit the proliferation of complex protocells with the extent of inhibition being most significant when a small fraction of protocells is eliminated during environmental cycling. Our work clarifies how the environment can shape the evolution and proliferation of complex protocells.

## 1. Introduction

The RNA world hypothesis, according to which an RNA-based life preceded the DNA and protein-based life on prebiotic earth, has been an important hypothesis regarding the origin of life. The abiotic synthesis of ribonucleotides [1,2,3] in simulated prebiotic scenarios, and the discovery of ribozymes [4,5,6] and their synthesis by in vitro evolution processes [7,8,9], are circumstantial evidence that have provided indirect support for this hypothesis. The possibility of the coexistence of RNA alongside amino acids [10] and lipids [11] on prebiotic earth has lead to the speculation of an updated version of the hypothesis: the RNA–lipid–peptide world. In this scenario, spontaneously formed lipid vesicles [12,13] can encapsulate RNA molecules, and such encapsulation turns out to be advantageous to both RNA molecules [14,15,16,17] and the vesicles that encapsulate them, as nucleotides are shown to have stabilizing effects on the lipid membranes [18]. RNA encapsulation also leads to the growth of lipid vesicles as it creates a difference in osmotic pressure between the vesicles containing RNA and empty vesicles, resulting in lipid transfer from the empty ones [19]. The presence of amino acids and small peptides is also advantageous as they, besides stabilizing the vesicle membranes [20], can turn them lipophilic [21], thereby causing further growth of the vesicles by lipid transfer from non-lipophilic vesicles. Large vesicles can divide when subject to external forces [22,23] and distribute their contents into daughter vesicles.

Computer simulations have shown that the evolution of functional RNA sequences within protocells offers several advantages [24,25,26,27,28] over their evolution in spatially open systems [29,30]. Ma and collaborators [26] have used Monte Carlo simulations to show how ribozymes created in open spatial systems can be engulfed by protocells, conferring a selective advantage to ribozyme-containing protocells that allows such protocells to survive and increase in number even as the ribozymes created in the open spatial system are gradually eliminated. Higgs and collaborators [27] have demonstrated that protocells can tolerate a lower replication rate as well as a lower replication fidelity of replicases without being over-run by selfish genetic elements. Even though these computational models provide important insights into the dynamics and evolution of RNA strands and protocells in a primordial RNA world, a plausible mechanism of formation of long and potentially functional RNA strands from basic building blocks is not discussed. These issues were addressed in [31], where we showed that both concatenation and template-directed primer extension in an environment subject to dry–wet cycling is essential for the creation of long and structurally complex RNA sequences from activated nucleotides. Our work raised the question of what might happen if such processes are confined within vesicular membranes. We have recently shown, using realistic, experimentally determined parameters, that a population of small vesicles encapsulating a small number of RNA molecules initially can evolve to a population consisting of large protocells containing multiple types of ribozymes via non-enzymatic rolling circle replication mechanism, protocell division and the preferential selection of vesicles with a higher number of RNA strands inside them [32]. Thus, a mixed RNA–lipid–peptide world can eventually lead to the possible emergence of a primitive protocellular life with protocells gradually increasing in complexity through the stochastic creation of a diverse set of ribozymes [32,33]. A key element, omitted in the above discussion, is that of the plausible environment for the origin and spread of primitive protocells. There has been much speculation on the ideal environmental conditions because it continues to be a critical factor in determining the viability of any protocell model for the origin and evolution of life.

**Figure 1:**
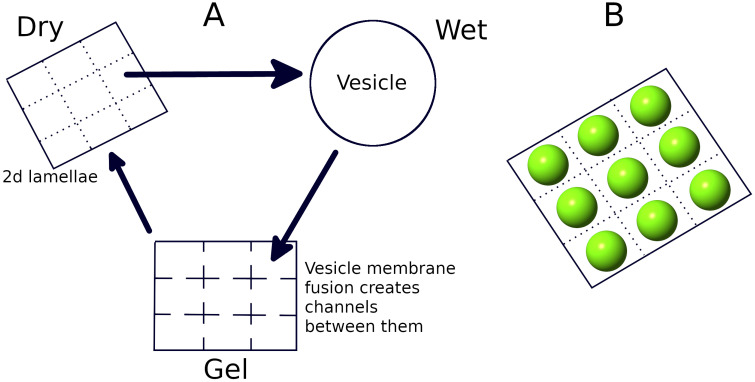
(**A**) Pictorial representation of the three phases that periodically occur in hot spring environments. Multilamellar structures can form on mineral surfaces from lipid molecules in the dry phase, one layer of which is represented as a 2D lattice containing sites for RNA polymerization. Each site can swell into a vesicle in the wet phase. In the gel phase, the vesicles become deposited on the 2D surface and their membranes start to fuse, creating channels between them that can allow for the long-range diffusion of large RNA strands. Subsequent to this stage, the multilamellar structure forms again in the next dry phase. (**B**) Three-dimensional (3D) representation of the formation of vesicles from the lamella in the wet phase.

There are two major hypotheses regarding a suitable environment for life’s origins: the hydrothermal vent hypothesis [34] and the hot spring hypothesis [35]. There have been significant efforts to analyze the efficacy of both these scenarios. The temperature gradient of submarine hydrothermal vents have been shown to be useful for RNA polymerization reactions in creating long RNA molecules [36,37,38,39]. Normally, in aqueous solutions, the average length of polymers depends on the polymerization ($K_{pol}$) and hydrolysis rates ($K_{hyd}$) as $L_{avg}∼\sqrt{K_{pol}/K_{hyd}}$ [40]. However, in the presence of a temperature gradient, molecules drift along the temperature gradient, which is a phenomenon known as thermophoresis [41]. It causes an influx of monomers, and as a result, the average length in this case also depends on the monomer influx rate (*J*) as $L_{avg}\propto \sqrt{J}$ [36]. Therefore, the temperature gradient of submarine hydrothermal vents can generate much longer RNA polymers compared to normal aqueous solutions [37]. Nevertheless, the hydrothermal vent hypothesis has certain disadvantages. The higher concentration of ionic solutes in seawater inhibits the formation of lipid vesicles and the encapsulation of polymers inside them [42]. Therefore, submarine hydrothermal vents, even though suitable for the formation of long polymers, are not the ideal environment for the formation of a protocellular life. Proponents of the alternative hot spring hypothesis argue that the region around hot springs/geothermal pools provides the ideal environment for the origin of life and evolution of protocells [35,43]. In addition to being rich in elements necessary for prebiotic chemistry [44,45], the periodic dry–wet cycles in such regions help in the creation of longer polymers [31,46]. Lipid molecules spontaneously formed in such regions can assemble into vesicles [47]. During the dry phase, vesicles fuse into multilamellar structures, and RNA monomers and oligomers become trapped between different layers of the lamella. The reduced water activity in this state aids phosphodiester bond formation and thereby helps in the synthesis of long RNA polymers at rates faster than the hydrolysis and degradation rates that break up such polymers. On the advent of the subsequent wet phase, the lamella swells into vesicles while still containing the RNA polymers, and as a result, the polymers become encapsulated in the vesicles [35,48]. An intermediate phase between the wet and dry phase results when the vesicle membranes start to fuse, thereby creating channels between them, allowing for the free long-range movement of both monomers and long polymers that were previously confined to a vesicle [35]. Figure 1 shows the pictorial representation of the three phases.

Along with laboratory-based experiments, recently, few experiments conducted near hot springs have provided confirmation of the spontaneous assembly of vesicles and formation of long RNA polymers in such locations [42,49,50]. All of these experimental findings, while providing strong empirical support of plausible prebiotic processes, are not adequate in explaining how primitive protocellular life could have evolved and spread in such regions. The experimental investigations in real hot spring environments are still at a stage of infancy, and it is indeed extremely difficult to conduct experiments of this scale in such inhospitable environments. In this context, computational models can be very useful in testing many of the key conceptual issues on protocell evolution in such environments that have been the subject of considerable speculation in the literature.

In our previous work [32], we considered the rolling circle replication process of circular RNA strands inside lipid vesicles. That choice was based on its effectiveness in maintaining the exponential growth of the number of RNA strands over a large temperature range [32]. If the hot spring hypothesis is true, a similar mechanism should be applicable even in such environments. Environmental factors such as wet–dry cycling and its impact on the physiology of the lipid lamella were not considered in our previous study. Here, we specifically test this key feature of the hot spring hypothesis of the origin of life by studying how phase transitions from the wet to gel-like and subsequently to a dry phase change the mobility of large RNA strands and consequently affect the evolution of the protocell population. While placing our model in a hot spring environment, we segregated the rolling circle replication and vesicle division processes in the dry and wet phases, respectively. This is because the lack of water inside the lamellae results in a kinetic trap [51], concentrating reactants and enhancing the rates of different types of polymerization reactions. In contrast, the wet phase causes vesicles to bud off from the lamella and increases their mobility, which in turn subjects them to external stress and shear forces that can lead to the division of larger vesicles. We considered a 2D lattice to represent the lamella and assumed the spontaneous formation of vesicles from different lattice sites during the wet phase, with vesicles formed at each site encapsulating the RNA molecules present at that site. In light of the increased mobility of long polymers during the gel phase, we included an extra process of long polymer diffusion from one site to another during this phase when the vesicles start to fuse.

We found that in the presence of dry and wet phase only, the initial population of protocells can evolve toward a state where protocells containing multiple types of ribozymes dominate. However, when the process of long polymer diffusion in the gel phase is taken into account, there is a significant slow down in the evolution of the protocell population, suggesting an inhibitory effect of gel phase diffusion. This effect is most pronounced when we consider degradation of entire protocells in the wet phase with small probabilities. The long polymer diffusion process in the gel phase then becomes the primary reason for the gradual decay and ultimately elimination of RNA strands, and as a result, the protocell population fails to evolve. On the other hand, the dry and wet phase alone or a dry, wet and gel phase that does not facilitate long polymer diffusion can sustain the evolution even in the presence of protocell degradation processes. Finally, in the presence of the dry and wet phase and absence of diffusion in the gel phase, we also observed a spatial expansion of the protocell population when initially one or very few of the sites contain circular templates, even when protocells are allowed to degrade in the wet phase. The growth of RNA strands inside those protocells followed by their subsequent division and occupation of neighboring sites by daughter protocells eventually lead to the outward spread of the protocell population with multiple ribozymes until it covers almost the entire lattice. Our work provides quantitative support for the viability of the hot spring environment in creating and sustaining an evolving population of protocells characterized by increasingly complex functionality.

## 2. Materials and Methods

According to the hot spring hypothesis [35,43], lipid molecules can periodically organize themselves to form three different types of structures determined by environmental conditions. In the dry phase, they can form a multilamellar structure on mineral surfaces. However, the dry phase is not completely dry to prevent any template-directed polymerization reactions [35]. It can be thought of as a semi-dry or semi-wet phase that allows template-directed polymerization processes to occur. The multilamellar structures act like kinetic traps confining reactant molecules and promoting polymerization reactions with activated nucleotides [31,52]. In the wet phase, local regions of that lamellae can swell into vesicles that can confine long RNA sequence fragments, and in the gel phase, the vesicle membranes fuse creating connected channels that can allow for the unrestricted movement of long polymers across the entire region. To simulate such a scenario, we start with a 2D square lattice (of size N×N=30×30) on a mineral surface representing the lamellae. Each square lattice site (of size Δx∼143 nm) provides a site for polymerization reactions to occur, while boundaries between lattice sites prevent the free movement of long polymers in the dry phase. In the wet phase, the lamellae from each site can bud off into different vesicles, thereby providing a confining environment that traps RNA polymers within the protocells. This is implemented by restoring the boundaries associated with each lattice site and thereby ensuring that each site acts as an independent protocell during the wet phase. In the gel phase, the disappearance of the boundaries between the lattice sites is mimicked by allowing for the free movement of long polymers across the entire lattice. We first start with one random circular ssRNA (denoted by *s*) template of length 200 nt [53] at each lattice site, at the beginning of a dry (lamellae) phase. Subsequently, we also consider an initial scenario where a single site has one or a small number of circular templates, while the remaining sites are devoid of any such templates. During the semi-dry phase, the molecules have very low mobility with only monomers and small oligomers (≤8 nucleotides) capable of diffusing freely across the lattice. The longer circular strands will remain fixed at their respective lattice sites.

### 2.1. Dry Phase: Non-Enzymatic Replication

At each lattice site inside the lamellae, a circular ssRNA can transform into a circular double-stranded RNA (dsRNA denoted by d in equations below) by attaching with a small complementary primer (∼8 nt) followed by non-enzymatic, template-directed extension of the primer. We assume the lipid membrane of the lamellae to be permeable to monomers and short oligomers [54] (≤8 nt) from the outside environment to maintain a continuous flow of primers so that the replication rates can be made independent of the primer concentrations. Upon becoming full length, the primer can extend further along the template by displacing its other end from its initial point on the template, thereby creating a hanging chain. When the hanging chain attains a length equal to the template, it cleaves and becomes separated from the circular dsRNA [32]. This process, called the rolling circle replication process [55,56,57], has been shown to be more effective [28] in producing many long ssRNA molecules and avoid the strand-separation problem associated with double-stranded RNA sequences. The non-enzymatic rate of creation of circular dsRNA from circular ssRNA and the rate of rolling circle replication of circular dsRNA is taken to be random for random templates and sampled from the distribution (see the Supplementary Information in [32] for details) $K=K_{0}e^{-a_{1}\times L-b_{1}\times \left(error/L\right)}\;h^{-1}$. This formula for *K* was determined from sequence-based simulations by fully extending a primer using the experimental primer extension rates [31,52,58] on an ensemble of random templates of different lengths, where we found that the replication rate depends on both the length (*L*) of the template and the relative amount of mismatch (error/L) produced during replication. We then fitted the data to obtain the values of the parameters $K_{0}=e^{-3.22}$, $a_{1}=0.005$ and $b_{1}=2.8$. The relative error (error/L) follows a normal distribution with mean μ=0.35 and standard deviation σ=0.0667. Therefore, from this formula, we can calculate the average and maximum replication rates for L=200 as $K^{avg}=0.0055\;h^{-1}$ and $K^{max}=0.00966\;h^{-1}$.

### 2.2. Dry Phase: Enzymatic Replication Using Ribozymes

The newly created, open-ended single strands (denoted by *l*) are diverse in terms of secondary structures [32] because of the error-prone nature of the replication process [52,58]. Therefore, we assume that a small fraction of them will attain catalytic capabilities associated with different types of ribozymes such as the replicase (*r*), cyclase (*c*), nucleotide synthase (*n*) and peptidyl transferase (*p*). Recent work by Dingle et al. [59] indicate that a relatively small number of random sequences are needed to completely sample the space of all RNA secondary structures (for a fixed sequence length), and among those, the ones that are found to be most frequent happen to be the ones that are found in nature. This suggests that sequences with complex secondary structures, that act as proxies for catalytic phenotypes, may not be too difficult to generate if adequate numbers of sequences are sampled. Since such a sampling process can be made possible through the rolling-circle mechanism that produces a large number of replicates, the assumption of a chance emergence of ribozymes may not be too unrealistic [32].

A replicase can catalyze the process of circular ssRNA to circular dsRNA formation by the template-directed primer extension mechanism and subsequently from circular dsRNA to an open-ended ssRNA by the rolling circle mechanism. At a site ij (i.e., the site labeled by the row-index *i* and column-index *j*; 1≤i≤N, 1≤j≤N), the rate of these processes would be $K_{fast}s_{ij}r_{ij}/V$ and $K_{fast}d_{ij}r_{ij}/V$ [9,32]), where we divide by a volume factor V=100 for each lattice site in unit of strand numbers to match the dimension of these second-order reactions. A cyclase (a type of ligase) can ligate the open ends of a non-catalytic open-ended ssRNA, creating a circular ssRNA at a rate $K_{fast}c_{ij}l_{ij}/V$. $K_{fast}$ was determined using the fastest replicase catalysed replication rate measured [9], for extending a primer by a single monomer. We used that rate to determine the average time taken to fully replicate a sequence of length *L* = 200, which is obtained after averaging over many sequences. The inverse of the average time yielded $K_{fast}=0.362$$h^{-1}$.

A nucleotide synthase can create new free nucleotides in a monomer-deficient system. Therefore, in the monomer-limited scenario that allows for the possibility of creation of a nucleotide synthase, we multiply each replication rate of site ij with a term $f_{ij}=\left(S_{ij}+bn_{ij}\right)/S_{ij}^{max}$, where $S_{ij}^{max}$ and $S_{ij}$ are the maximum and instantaneous number of monomers in a site in units of 200 mers and *b* is the number of monomers in units of 200 mers that a single nucleotide synthase can create (we set b=1 and $S_{ij}^{max}=80$ in the simulations). Finally, a peptidyl transferase can join free amino acids that are assumed to be present in the system to create small peptide chains. During the wet phase, when the lamellae from different lattice sites swell into vesicles, such small peptide chains can bind with the lipid membrane of the vesicles and turn them into lipid-attracting membranes, thereby causing them to grow in size [21] and increasing the threshold volume ($V_{T}^{ij}\to V+Qp_{ij}$, where *Q* is the strength of a peptidyl transferase and measures the amount by which the threshold volume increases per peptidyl transferase ribozyme present in the protocell beyond which the vesicle divides into two daughter protocells. Finally, all types RNA molecules inside the lamellae can degrade at a certain rate (*h*). These reactions form the basis of our coarse-grained model and can be represented as the following set of equations (the definition and value of each term in the equations are provided in Table 1):

(1) $$\dot{s_{ij}}=K_{fast}\frac{c_{ij}l_{ij}}{V}-\sum_{\nu =1}^{s_{ij}}K_{\nu }^{ij}f_{ij}-K_{fast}\frac{r_{ij}s_{ij}f_{ij}}{V}-hs_{ij}$$

(2) $$\dot{d_{ij}}=\sum_{\nu =1}^{s_{ij}}K_{\nu }^{ij}f_{ij}+K_{fast}\frac{r_{ij}s_{ij}f_{ij}}{V}-hd_{ij}$$

(3) $$\begin{array}{c} \dot{l_{ij}}=\left(1-P_{r}-P_{c}-P_{n}-P_{p}\right)\sum_{\nu =1}^{d_{ij}}K_{\nu }^{ij}f_{ij}+(1-P_{r}-P_{c}\\ -P_{n}-P_{p})K_{fast}\frac{r_{ij}d_{ij}f_{ij}}{V}-K_{fast}\frac{c_{ij}l_{ij}}{V}-hl_{ij} \end{array}$$

(4) $$\dot{r_{ij}}=\sum_{\nu =1}^{d_{ij}}P_{r}K_{\nu }^{ij}f_{ij}+P_{r}K_{fast}\frac{r_{ij}d_{ij}f_{ij}}{V}-hr_{ij}$$

(5) $$\dot{c_{ij}}=\sum_{\nu =1}^{d_{ij}}P_{c}K_{\nu }^{ij}f_{ij}+P_{c}K_{fast}\frac{r_{ij}d_{ij}f_{ij}}{V}-hc_{ij}$$

(6) $$\dot{n_{ij}}=\sum_{\nu =1}^{d_{ij}}P_{n}K_{\nu }^{ij}f_{ij}+P_{n}K_{fast}\frac{r_{ij}d_{ij}f_{ij}}{V}-hn_{ij}$$

(7) $$\dot{p_{ij}}=\sum_{\nu =1}^{d_{ij}}P_{p}K_{\nu }^{ij}f_{ij}+P_{p}K_{fast}\frac{r_{ij}d_{ij}f_{ij}}{V}-hp_{ij}$$

We solve the fully stochastic version of these seven equations [32] in our simulations (more details are given in the Supplementary Information File). As a consequence of these above-mentioned reactions (which are favored by the low amount of water in the dry phase), the number of strands at each lattice site can grow and eventually be encapsulated in a vesicle forming at that site from the lamella during the subsequent wet phase.

### 2.3. Wet Phase: Protocell Division

The presence of a high amount of water in the wet phase will cause the vesicles to undergo Brownian motion, thereby making them highly mobile. For example, a vesicle of diameter 60 nm can move across our entire lattice in less than 10 s [60]. Such high mobility of the vesicles can make them collide with sharp rock edges or move through rock pores. They can even be subjected to shear and stress due to the turbulent flow of the water in which they are immersed. Such forces can induce the division of larger vesicles [22,23]. In our model, when the number of RNA molecules inside such a vesicle exceeds a certain threshold (depending on the number of peptidyl transferase ribozymes inside it), the vesicle will divide into two daughter vesicles. As division causes an increase in the total surface area (26% for perfectly spherical vesicles), we assume the elimination of another vesicle (chosen from one of the eight nearest neighbors) to provide the lipid molecules needed for that extra surface area. Selection is introduced by imposing the condition that vesicles with fewer RNA strands inside them are more likely to be eliminated. Therefore, we take the elimination probability of a neighboring vesicle to be proportional to the difference between the number of RNA strands inside it and in the dividing vesicle.

### 2.4. Gel Phase: Long Strand Diffusion

In the dry phase, there are no vesicles, and long RNA strands on specific site are immobile even though monomers and short oligomers can diffuse freely. During the transition from the wet to the dry phase, as the excess water starts to dry up due to increasing temperatures, creating a gel phase, it causes the vesicle membranes to fuse, creating channels from one vesicle compartment to another on the multilamellar lattice. This phase allows for the increased mobility of even large RNA strands, thereby allowing for their potential dispersal to different regions of the lattice. The diffusion coefficients of the long strands can be related to their hopping probabilities from one lattice site to a neighboring site by the equation [61]:

(8) $$D=\frac{P_{hop}\times {\left(\Delta L\right)}^{2}}{dim\times \Delta t}$$

The diffusion of molecules from one site to another also depends on the difference in the number of strands between them. The molecules from the central site are more likely to diffuse to the neighbor that is relatively empty. The size of the lattice site is ΔL=143 nm. Therefore, for a time step size Δt=0.008 h, the maximum diffusion coefficient (for $P_{hop}=1$) in 2D (dim = 2) would be $D∼1.28\;\mu m^{2}\;h^{-1}$, which is ∼$10^{5}$ times smaller than the diffusion coefficients of 200 mer RNA strands (single and double stranded) if they were present freely outside the vesicles in the fully hydrated wet phase [62,63]. Although experimentally, it is found that double strands have a lower diffusion coefficient than single strands, we found that our results do not differ even if we consider different hopping probabilities for the circular dsRNA molecules. We take the duration of the dry, wet and gel phase to be equal to 8 h each so that they can appear periodically for each day of length 24 h. The simulations were run for many such days using periodic boundary conditions on the lattice.

## 3. Results

### 3.1. Effect of the Individual Phases

**Figure 2:**
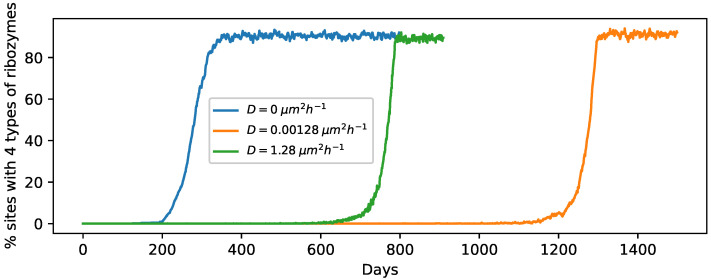
Time evolution plots (1 trial each) showing the percentage of sites containing all 4 types of ribozymes for $D=0\;\mu m^{2}\;h^{-1}$ (blue); $D=0.00128\;\mu m^{2}\;h^{-1}$ (orange) and $D=1.28\;\mu m^{2}\;h^{-1}$ (green) starting from 1 circular ssRNA per site initially.

We first simulated the evolution of RNA strands and protocells in the dry and wet phase after inactivating the gel phase in order to understand the dynamics in the absence of long-strand diffusion. Such a scenario also provides a benchmark for evaluating the impact of diffusion in the gel phase when it is subsequently switched on. We find that the strands in a few sites which happen to contain faster replicating circular templates initially increase and become encapsulated in vesicles during the subsequent wet phase. However, at most other sites, the initial circular templates have low–medium replication rates as a result of which new RNA strands including ribozymes are not produced at a rate that is faster compared to the degradation process. As a consequence, those sites become devoid of RNA strands. This results in the concentration of a large number of strands at a few sites, while most of the remaining sites become empty. Eventually, as the vesicles originating from those few sites start to divide when the threshold volume is attained, the newly created daughter vesicles occupy the empty nearest neighbor sites. Through this vesicle division process, the RNA molecules gradually spread over the entire lattice, and we find that ∼90% sites (and subsequent protocells) contain all four types of ribozymes at equilibrium (Figure 2, D=0). The evolutionary push toward the dominance of protocells with all four types of ribozymes comes from the formation of a cooperative network between these four types of ribozymes [32]. The Supplementary Video S1 shows the evolution of different types of RNA strands and ribozyme diversity across the entire lattice (more details about the video are given in the Supplementary Information).

When the gel phase is switched on, allowing for the diffusion of long RNA polymers in that phase, the evolution toward equilibrium is much slower. The plots for the percentage of sites with four types of ribozymes vs. time for two diffusion coefficients are shown in Figure 2. Unlike the *D* = 0 case, the time at which the curve starts rising steeply from 0 occurs at different times for different trial runs even for the same diffusion coefficient. Nevertheless, we observed that the time evolution for non-zero *D* values is still much slower than the *D* = 0 case across all trials. We therefore show the curves for only one trial for each diffusion coefficient in Figure 2. Video S2 shows the proliferation of RNA strands across the entire lattice when diffusion is allowed in the gel phase (for $D=1.28\;\mu m^{2}\;h^{-1}$). The dispersal of long RNA strands can impede the growth of RNA strands at a specific site, delaying the protocell division process and the eventual proliferation of daughter protocells to neighboring sites. For example, if a replicase emerges at a site having a faster replicating template initially, then it would speed up the replication process at this site by many folds, thereby increasing the likelihood of the formation of new ribozymes. However, if such a replicase responsible for the rapid growth of RNA strands at a site were to disperse to another site in the gel phase, it would limit the subsequent growth of RNA strands and the stochastic creation of ribozymes at the original site. Moreover, the effectiveness of the replicase at the new site might be hindered by the presence of fewer circular templates and/or other types of ribozymes. Similarly, if a cyclase, responsible for catalyzing the formation of new circular templates on which a replicase can act to speed up the replication process, diffuses to another site, it can significantly slow down the creation of new strands at the original site. Even though long RNA strand diffusion in the gel phase has an inhibitory effect on the evolution and proliferation of protocells with increasing complexity, given sufficient time, complex protocells containing multiple ribozymes can still proliferate and occupy ∼90% sites, as evident from the equilibrated values shown by the orange and green curves in Figure 2.

### 3.2. Relocation of Protocells in the Wet Phase

As mentioned in the methods section, the presence of water in the wet phase will cause the vesicles to undergo rapid Brownian motion. Therefore, a vesicle which originates from a site on the lattice will not deposit on the same site after the wet phase. The Brownian motion of vesicles during each wet phase will effectively result in random relocation of the vesicles on the lattice. To account for this phenomenon, we randomly shuffle the vesicle positions on the lattice at the beginning of each wet phase. Protocell division occurs after reshuffling in those protocells where the number of RNA strands has crossed the threshold volume. Video S3 shows the evolution of the protocell population in this scenario. When such a relocation of protocells is allowed, the percentage of sites with four types of ribozymes takes somewhat longer to saturate (figure not shown), but not as long as it takes to reach equilibrium in the presence of gel phase strand diffusion. However, the equilibrium abundance of protocells with four types of ribozymes drops to ∼72%. This happens because in the *absence* of the relocation, a few initial vesicles with faster replicating strands cross the threshold volume, leading to vesicle division, which spreads some of their contents encapsulated in newly formed daughter protocells to neighboring sites. This process happens from a few locations on the lattice, and as the process continues, it leads to the formation and outward growth of clusters of vesicles containing all four ribozymes. This happens because dividing vesicles located on the edge of the cluster are more likely to contain larger numbers of ribozymes with one of the daughter protocells more likely to eliminate vesicles neighboring the boundary of the cluster (that have relatively fewer ribozymes) than those in the interior or edge of the cluster (that have a relatively larger number of ribozymes). Eventually, the spread of functionally complex protocells occurs through the merging of these expanding clusters. This is evident from Video S4, which tracks the evolution of protocells containing three and four ribozymes, as well as dividing protocells (see the Supplementary Information for details). However, when protocells are relocated in the wet phase, the cluster formation of protocells containing a larger number of ribozymes is inhibited, and the spread of complex protocells occurs due to the creation of such protocells, initially sparsely but eventually more uniformly, across the spatial landscape. This can be seen in Video S5, which reveals the contrasting evolutionary dynamics in comparison to the one depicted in Video S4.

### 3.3. Degradation of Entire Protocells in the Wet Phase

**Figure 3:**
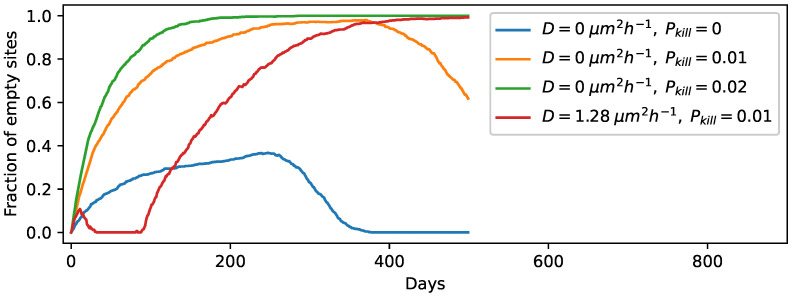
Time evolution of the fraction of empty sites for $D=0\;\mu m^{2}\;h^{-1}$ with no protocell degradation in the wet phase; $D=0\;\mu m^{2}\;h^{-1}$ when protocells degrade with probability $P_{kill}=0.01$; $D=0\;\mu m^{2}\;h^{-1}$ when protocells degrade with probability $P_{kill}=0.02$; $D=1.28\;\mu m^{2}\;h^{-1}$ when protocells degrade with probability $P_{kill}=0.01$.

So far, we have assumed that the protocells remain intact during the wet phase, but in reality, some protocells will degrade in every wet phase. To account for this effect, we include the process of protocell destruction in our simulation at the beginning of every wet phase. This is likely to inhibit the spread of protocells of increasing complexity in a manner that is dependent on the protocell killing probability. Each protocell can degrade along with its contents with a probability $P_{kill}$ with the location of the degraded protocell remaining empty until it is occupied by another protocell as a result of diffusion or a division event at a neighboring site. We first consider the case without strand diffusion in the gel phase, with our simulations being initiated with one circular template per site. We varied $P_{kill}$ and found that the protocell population can evolve up to a threshold $P_{kill}\leq 0.01$, above which all RNA strands die out across the lattice. This threshold increases to $P_{kill}\leq 0.02$ when we start with five random templates per site. However, when the strand diffusion process is present, even for diffusion coefficients as low as $3.2\times 10^{-5}$ μ$m^{2}$h${}^{-1}$ (with $P_{kill}=0.01$), the protocells containing RNA strands (even excluding ribozymes) can no longer be sustained in the population. The videos showing the temporal evolution for $P_{kill}=0.01$ in the absence and presence of diffusion in the gel phase (corresponding to the orange and red curves in Figure 3) are provided in the Supplementary Information (see Videos S6 and S7 respectively). We arrived at a similar result even when we started the simulations with five templates per site. Therefore, consistent with the results of previous sections, the strand diffusion process in the gel phase is found to be counter-productive for the proliferation of protocells containing functionally complex components. Figure 3 shows the plots for the fraction of empty sites with time for three different $P_{kill}$ values in the absence and presence of diffusion in the gel phase. We also checked the time evolution for diffusion coefficients that are 10-fold higher (D=12.8 μm${}^{2}$h${}^{-1}$) and for experimental diffusion coefficients in hydrated conditions, which corresponds to the well-mixed limit in our setup. Even for those scenarios, the behavior is similar to that observed for $D=1.28\;\mu m^{2}\;h^{-1}$ in Figure 3. Protocell relocation in the wet phase, however, does not hinder the evolution as much as gel phase strand diffusion does, because the protocells with all four types of ribozymes still dominate with an abundance of ∼65% even when the protocells are allowed to degrade with probability $P_{kill}=0.01$.

**Figure 4:**
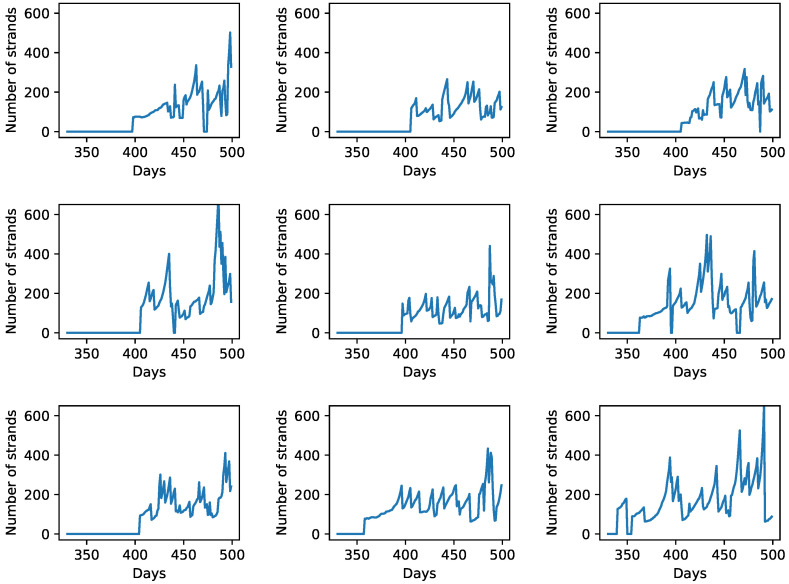
Total number of RNA strands vs. time for a site having a low $K_{rep}$ value of the initial template and its eight neighboring sites for the case when protocells can degrade in the wet phase with probability $P_{kill}=0.01$. In this figure, the plots for those 9 sites are arranged in a manner that is identical to their arrangements on the lattice; i.e., the plot in row 2, column 2 corresponds to the central site and the sub-plots surrounding it correspond to its 8 neighbors.

**Figure 5:**
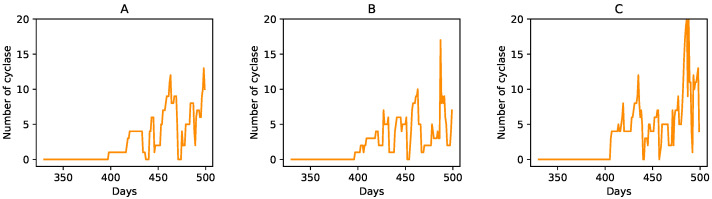
Number of cyclases vs. time for sites corresponding to panels in (**A**) row 1, column 1, (**B**) row 2, column 2 and (**C**) row 2, column 1; shown in Figure 4.

We also plot the total number of strands vs. time of a random site and its eight nearest neighbors for the case of *D* = 0 and $P_{kill}=0.01$ (Figure 4). As evident from the figure, during this time period, the total number of strands of a site goes to zero six times (counting across all the nine sites) as a result of the protocell degradation process. The large fluctuations in the number of RNA strands observed in the plots is a consequence of the protocell division process when a parent protocell divides upon reaching the threshold volume, and its strands are divided roughly equally between its two daughter protocells. For example, the central site (the sub-plot in row 2, column 2 of Figure 4), which does not contain any strands initially, acquires strands by the protocell division process from either the protocell at the bottom center panel (row 3, column 2 of Figure 4) or the protocell at the bottom right panel (row 3, column 3 of Figure 4), both of which divide in the previous wet phase. Tracking the temporal variation in a specific ribozyme (such as the cyclase) across these nine sites reveals features that are not evident from Figure 4. Not surprisingly, the total number of cyclase goes to zero more often than the total number of strands. However, this often happens as a consequence of the asymmetric distribution of RNA strands across the two daughter protocells following a protocell division event. To highlight this fact, Figure 5 shows the temporal variation in the number of cyclase for three of the nine sites depicted in Figure 4. In Figure 5B (central site), when the cyclase number goes to zero from six just after t=450 days, the number of cyclase at a neighboring site (Figure 5C) becomes six, indicating that one of the daughter protocells of the central site that acquired all the cyclases after division has occupied a neighboring site, while the other daughter cell that does not contain any cyclase has replaced the parent protocell at the central site.

### 3.4. Spatial Expansion of Protocell Population

**Figure 6:**
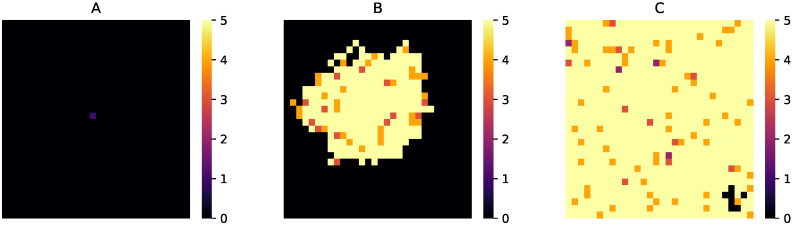
Expansion of protocell population from only one site containing 5 circular ssRNA templates initially. (**A**) Initial stage; (**B**) intermediate stage (on the 300th day) and (**C**) when the percentage of sites with all 4 ribozymes becomes 90%. Color values: Black: 0→ no strands, Purple: 1→ contains only non-enzymatic strands, Magenta: 2→ contains 1 type of ribozyme, Orange: 3→ contains 2 types of ribozymes, Dark Yellow: 4→ contains 3 types of ribozymes, Light Yellow: 5→ contains 4 types of ribozymes.

Given how a protocell can divide and its daughters can occupy a nearest neighbor site, we wanted to ascertain if the protocell population can proliferate across the entire lattice, starting from an initial state where only a few sites contain the initial templates. We first checked for the case with strand diffusion in the gel phase (D=0 μm${}^{2}$h${}^{-1}$); protocell relocation and degradation processes were all absent. We varied the fraction of initial sites that contain a circular ssRNA and found that the protocell population can evolve and spread to the entire lattice if at least 2% of the initial sites contain a template. Interestingly, even if we start with only one non-empty site (out of 900 sites), the protocell population can still evolve and spread provided that the single site contains at least five circular templates with $K_{rep}$ values taken from the distribution. Figure 6 shows the population evolution for this case at three different stages. Next, we included the strand diffusion process (with a small D=0.00128  $\mu m^{2}\;h^{-1}$), which for our lattice site dimension ΔL = 143 nm and the gel phase duration $T_{gel}$ = 8 h indicates that each strand can move to a neighboring site only once during each day with probability $P_{hop}=1$) in the gel phase. We found that at least 20% of the sites need to contain at least five templates for the protocell population to evolve and expand spatially. Otherwise, the entire RNA population gradually dies out. In the case of only *one* non-empty site, even starting with 50 templates is inadequate to ensure the proliferation of protocells when strand diffusion is present. Therefore, strand diffusion in the gel phase can prevent the gradual spatial expansion of a functionally complex protocellular population. However, when the protocell relocation process in the wet phase was allowed to occur instead (with strand diffusion turned off), 2% of the initial sites containing one template each were able to evolve and expand, indicating that this process does not hamper spatial expansion. Finally, to check if initially starting with templates in fewer number of sites can lead to the evolution and expansion of the protocell population even when protocells can degrade in the wet phase, we varied both the fraction of non-empty sites and the number of initial templates in them. We found that expansion is possible when there are at least 10% non-empty sites with at least five random templates in each of them, and such expansion is possible even in the presence of the protocell relocation process.

## 4. Conclusions

Any compelling model of protocell evolution in a primordial RNA world must account for plausible environmental conditions in which such processes occurred and address the constraints imposed on the evolutionary processes by such conditions. The hot spring hypothesis of the origin of life provides an interesting test-bed to study evolutionary processes and assess the viability of such processes in aiding the formation, growth, division and eventual proliferation of protocells of increasing functional complexity. The ecological niche around hot springs when subject to dry–wet cycling provides suitable conditions for the formation of long RNA polymers which can then be encapsulated into vesicles. The encapsulation of RNA sequences by protocells and the replication of RNA via the rolling circle mechanism can lead to rapid growth of number of RNA strands and also increase the likelihood of the creation of ribozymes of diverse functionality [32]. In this paper, we examined how the coupling between the hot spring environment and protocell evolution affects the proliferation of protocells containing multiple ribozymes. We showed how the interplay between the dry and the wet phase, where the former helps in polymerization reactions and the latter helps in protocell formation and division, can be critical for spreading the innovation in the form of ribozymes that are produced initially in a few protocells. In absence of dry–wet cycling, such innovations are localized in a few regions and are likely to be lost in the course of evolution.

The hot spring hypothesis emphasizes the importance of the gel phase when protocells compartments can exchange molecules with each other by the strand diffusion process through their fused membranes. A question that can be raised in the light of our analysis is whether a gel phase characterized by the diffusion of long RNA sequences is essential for the spatial proliferation of functionally complex protocells containing multiple ribozymes. It has been argued [35] that the usefulness of the gel phase stems from its ability to facilitate communal evolution. The fusion of the protocellular membranes leads to the creation of a single connected, albeit dispersed, community of RNA strands that can benefit from innovation in RNA sequences (such as the appearance of a new ribozyme) that may appear in one region of the community. However, for such innovations to be effective, they need to be replicated and spread across the regions. Moreover, the advantage of communal evolution is considerably diminished if the communal structure is periodically destroyed due to environmental cycling. Our results indicate that the diffusion of long RNA strands across fused protocell compartments in the gel phase inhibits the proliferation of functionally diverse protocells with the effect being most detrimental when the process of protocell degradation is taken into account.

Experimental studies [35,48,51] have already revealed the formation of multilamellar structures in the gel phase and the budding of lipid vesicles containing DNA. The effectiveness of the rolling circle replication process [28,32] used in our model to generate new RNA strands, especially in the presence of a polymerase ribozyme, has recently been demonstrated [64]. Certain aspects of the hot spring hypothesis captured in our computational model, such as the inhibitory effect of long RNA strand diffusion in the gel phase, can be subjected to experimental validation in the lab. We therefore hope our work will stimulate further experimental investigations in the lab by simulating conditions prevalent near geothermal hot springs and subject our model of protocell evolution to rigorous testing. The puzzle of origin of life is still far from being solved, but many pieces of the puzzle are beginning to fall into place. We believe that conceptual advances accompanied by new insights from experiments will enable us to make significant progress in understanding this challenging topic.

## Figures and Tables

**Table 1:** Details of each term used in Equations (1) to (7).

Term	Definition	Value
$s_{ij}$	Number of circular ssRNA at site (i,j)	variable
$d_{ij}$	Number of circular dsRNA at site (i,j)	variable
$l_{ij}$	Number of open-ended ssRNA at site (i,j)	variable
$r_{ij}$	Number of replicase at site (i,j)	variable
$c_{ij}$	Number of cyclase at site (i,j)	variable
$n_{ij}$	Number of nucleotide-synthase at site (i,j)	variable
$p_{ij}$	Number of peptidyl-transferase at site (i,j)	variable
$K_{fast}$	Replicase-catalyzed replication rate, cyclase-catalyzed new circular ssRNA creation rate	$0.362\;h^{-1}$
$K_{\nu }^{ij}$	Non-enzymatic replication rate of $\nu^{\prime }th$ template (circular ssRNA or dsRNA) at site (i,j)	variable
*V*	Reference volume of each lattice site	100
$f_{ij}$	Monomer dependent rate reduction factor at site (i,j)	$\left(S_{ij}+bn_{ij}\right)/S_{ij}^{max}$
$S_{ij}$	Instantaneous number of monomer at site (i,j) in unit of 200 mers	variable
*b*	Number of monomers created by a nucleotide- synthase in unit of 200 mers	1
$S_{ij}^{max}$	Maximum number of monomer at site (i,j) in unit of 200 mers	80
*h*	Degradation rate of each strand	$0.0008\;h^{-1}$
$P_{r},\;P_{c},$ $P_{n},\;P_{p}$	Replicase, Cyclase, Nucleotide-synthase and Peptidyl-transferase creation probabilities respectively	0.03, 0.03, 0.03, 0.03
$V_{T}^{ij}$	Threshold volume for division of vesicle created from site (i,j)	$V+Qp_{ij}$
*Q*	Strength of a peptidyl-transferase in increasing the threshold volume for division	20

## Data Availability

The data sets that support the findings of this study are available from the corresponding author upon reasonable request. The main computer code used to generate this data, is available at the following https://github.com/suvamroy/Codes/blob/master/Hot_spring_model/Main_code.py link (accessed on 5 August 2022).

## Supplementary Materials

The following supporting information can be downloaded at: https://www.mdpi.com/article/10.3390/life12081227/s1.
